# Rocky Mountain Conference on Bioinformatics Celebrates 10 Years

**DOI:** 10.1371/journal.pcbi.1003076

**Published:** 2013-05-30

**Authors:** Lawrence E. Hunter

**Affiliations:** Center for Computational Pharmacology & Computational Bioscience Program, University of Colorado Denver, Aurora, Colorado, United States of America; Princeton University, United States of America

The annual Rocky Mountain Conference on Bioinformatics, better known just as “Rocky,” celebrated its tenth meeting in 2012. Rocky has a unique approach in bioinformatics, bringing attention to new scientists and working hard to facilitate the creation of new collaborations and relationships. Set in the spectacular scenery of the Roaring Fork Valley, not far from world-renowned Aspen, Colorado, Rocky offers a world-class locale to accompany great science. Although many of the 127 attendees were from the Rocky Mountain region, scientists came from as far away as Egypt and Korea to join the meeting. Rocky runs two-and-a-half days, organized with keynote addresses bracketing lightning talks and ski breaks.

The 2012 keynotes included well-known academic stars Chris Mungall and Olga Troyanskaya, senior thinkers from industry Kirk Jordan and Alex Stewart, and rising young informaticians James Costello, Yuval Itan, and Chris Miller. Miller described novel metagenomic methods that he used to characterize microbiome communities in some of the most polluted sites on earth, describing how to increase sensitivity for detection of rare organisms, and characterizing the surprisingly rich ecosystems found in extremely toxic environments. Itan presented a method for building a network of biological (rather than strictly genetic) relationships among all human genes, then applied graph algorithms to discover disease-causing alleles at genomic scale. He also described impressive experimental validations of his predictions. Costello discussed his analysis of the DREAM competition, where dozens of groups analyzed blinded data regarding gene regulatory networks and genetic determinants of drug response. He demonstrated the value of multiple, independent approaches to difficult prediction problems, and inverted the original goal of the competition to provide insights into the differential predictive power of different sorts of data. Mungall described recent advances in biomedical ontology, which increasingly support complex logical inference, providing complementary information to the traditional statistical analysis of genomic data. Troyanskaya presented a plethora of recent results from her lab, including a remarkable informatics approach to deconvolving cell type– and tissue–specific contributions to gene function and regulation. Jordan described the latest innovations in IBM's high performance computing division, and showcased some impressive applications in medicine, physiological modeling, and sequence analysis. Stewart described SomaLogic's impressive technology that quantitatively assays the abundance of more than 1,000 proteins, demonstrated its clinical utility in stable coronary heart disease, and talked about the informatics challenges involved.

In addition to these keynote addresses, 46 scientists from around the world presented 7-minute talks on their research. The purpose of the lightning talks is to briefly introduce the work of each scientist, facilitating more detailed conversations during the abundant free time provided. The topics of the lightning talks spanned a huge range of bioinformatics, from an ENCODE analysis shedding light on the plasticity of replication timing dependent on chromatin structure to an ontological analysis of the discourse structure of scientific journal articles. Topics ranged from phylogenetics to drug response in cancer, and the methods described covered an enormous range, including, for example, mechanistic modeling, Bayesian inference, text mining, and phylogeography.

Several of the presenters were giving their first public scientific talk. Rocky is proud of its history of giving the first stage to many young investigators who have gone on to impressive careers. Given the large number of speakers, it is relatively easy to get a chance to present relevant work. There was even one undergraduate researcher reporting on his (impressive!) results. The conference environment sets a relaxed environment for graduate students, postdocs, and other early stage researchers to interact with senior scientists. A remarkably large number of Rocky participants report that new interactions and collaborations arose from their attendance at the meeting, and nearly all say the experience exceeded their expectations.

Next year's Rocky is already in preparation, and will be held at the Viceroy Hotel in Snowmass, CO, December 12–14, 2013. The later dates should help with the snow coverage, and the scientific agenda promises to once again be outstanding. We hope to see you there.

